# Incidence and risk factors for clinically confirmed secondary bacterial infections in patients hospitalized for coronavirus disease 2019 (COVID-19)

**DOI:** 10.1017/ice.2023.27

**Published:** 2023-10

**Authors:** Hiromichi S. Park, Caitlin M. McCracken, Noah Lininger, Cara D. Varley, Monica K. Sikka, Christopher Evans, Angela Holly Villamagna, Jina T. Makadia, Jessina C. McGregor

**Affiliations:** 1 Division of Infectious Diseases, Department of Medicine, Oregon Health & Science University, Portland, Oregon; 2 Department of Pharmacy Practice, College of Pharmacy, Oregon State University, Corvallis, Oregon; 3 Program in Epidemiology, Oregon Health & Science University–Portland State University School of Public Health, Portland, Oregon

## Abstract

**Objective:**

The true incidence and risk factors for secondary bacterial infections in coronavirus disease 2019 (COVID-19) remains poorly understood. Knowledge of risk factors for secondary infections in hospitalized patients with COVID-19 is necessary to optimally guide selective use of empiric antimicrobial therapy.

**Design:**

Single-center retrospective cohort study of symptomatic inpatients admitted for COVID-19 from April 15, 2020, through June 30, 2021.

**Setting:**

Academic quaternary-care referral center in Portland, Oregon.

**Patients:**

The study included patients who were 18 years or older with a positive severe acute respiratory coronavirus virus 2 (SARS-CoV-2) PCR test up to 10 days prior to admission.

**Methods:**

Secondary infections were identified based on clinical, radiographic, and microbiologic data. Logistic regression was used to identify risk factors for secondary infection. We also assessed mortality, length of stay, and empiric antibiotics among those with and without secondary infections.

**Results:**

We identified 118 patients for inclusion; 31 (26.3%) had either culture-proven or possible secondary infections among hospitalized patients with COVID-19. Mortality was higher among patients with secondary infections (35.5%) compared to those without secondary infection (4.6%). Empiric antibiotic use on admission was high in both the secondary and no secondary infection groups at 71.0% and 48.3%, respectively.

**Conclusions:**

The incidence of secondary bacterial infection was moderate among hospitalized patients with COVID-19. However, a higher proportion of patients received empiric antibiotics regardless of an identifiable secondary infection. Transfer from an outside hospital, baseline immunosuppressant use, and corticosteroid treatment were independent risk factors for secondary infection. Additional studies are needed to validate risk factors and best guide antimicrobial stewardship efforts.

The coronavirus disease 2019 (COVID-19) pandemic has produced significant global morbidity and mortality. Illness and severity can vary from mild cases to severe respiratory failure manifested as acute respiratory distress syndrome (ARDS).^1,2^ The disease course can also be complicated by diverse types of secondary infections ranging from bacterial pneumonia to opportunistic fungal infections.^2,3^ However, the incidence and prognosis of secondary infections in COVID-19 are poorly understood. Community-onset infections have been reported at overall low incidence ranging from 1.18% to 3.5%.^4,5^ In contrast, hospital-acquired secondary infections have been reported at higher incidence, ranging from 11.9% up to 53%.^1,6,7^ Meta-analyses have reported a pooled incidence of community-onset secondary infections following COVID-19 at 19% and hospital-acquired infections at 24%, where others report relatively lower incidence of secondary infection of 8.6%.^8,9^ The considerable heterogeneity among studies is likely due to variability in setting, testing methods, and case definitions, which frequently include only culture based or molecular testing as their definition for secondary infection without clinical criteria.^8,9^ In addition, there are significant challenges in distinguishing secondary bacterial infections from severe acute respiratory coronavirus virus 2 (SARS-CoV-2). Secondary infections are reported in a small proportion of COVID-19 infections; however, earlier studies have reported up to 98% of patients received antibiotics.^1–5,8^ In this study, we measured the incidence of secondary infections among hospitalized patients with symptomatic COVID-19 and identify independent risk factors for secondary infections.

## Methods

### Study design and setting

We performed a retrospective cohort study of patients with symptomatic COVID-19 hospitalized at Oregon Health & Science University hospital (OHSU) between April 15, 2020, and June 30, 2021. OHSU is a quaternary-care, 576-bed academic medical center in Portland, Oregon. As the only academic medical center in the state, OHSU serves Oregon, southern Washington, northern California, Idaho, Montana, and Alaska. The first case of COVID-19 in the state of Oregon was identified on February 19, 2020, the first case at OHSU on March 8, 2020, and an executive stay-at-home measure implemented on March 23, 2020.^10–12^ The OHSU Institutional Review Board approved this study.

Using an electronic health record data repository, we identified patients 18 years or older with a positive nasopharyngeal SARS-CoV-2 PCR test up to 10 days prior to the day of admission. We confirmed symptomatic COVID-19 by medical record review for supplemental oxygen requirements and documentation of COVID-19 as the principal reason for hospitalization. Asymptomatic or recovered individuals that were admitted for reasons other than COVID-19, and those who had only positive antigen or serologic tests, were excluded. Patients with symptoms limited to fever or cough, who did not have indications for hospitalization related to COVID-19, were excluded.

### Primary outcome definition

Our primary outcomes were secondary bacterial pneumonia or bacteremia. Our bacterial pneumonia definition was adapted from the Centers for Disease Control and Prevention’s National Healthcare Safety Network (NHSN) definitions for ventilator- and non–ventilator- associated pneumonia and focused on 3 major criteria: clinical, radiographic, and microbiologic.^13^ All patients’ medical records were manually reviewed to assess these criteria. Radiographic evidence was evaluated specifically to differentiate between COVID-19 and bacterial pneumonia. Bacteremia was identified as a positive blood culture by a known pathogen with compatible clinical features. Definitions are further specified in Box 1.

**Box 1.**
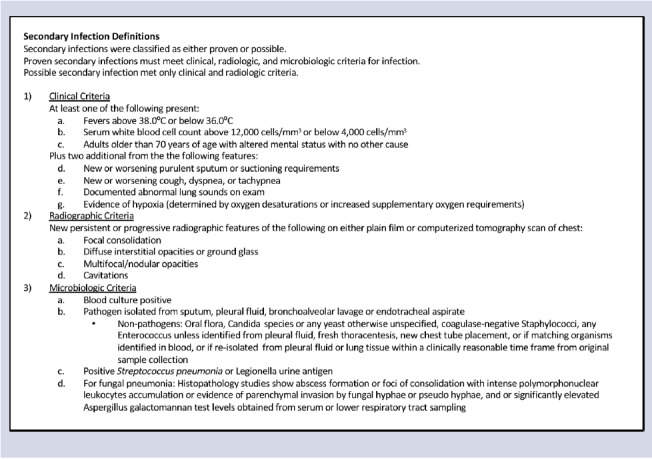
Secondary Infection Case Definition

We defined secondary bacterial pneumonia as a proven secondary infection if all 3 major criteria were met. We defined secondary bacterial pneumonia as a possible case if clinical and radiographic criteria were met but not microbiologic criteria. We categorized secondary infections as community onset (onset within 48 hours of admission), or hospital acquired (onset > 48 hours after admission), considering whether the patient was transferred from an outside facility. Time of onset was defined as time of specimen collection for proven secondary infections and the time of clinical onset for possible infections medical record review was initially performed by 2 investigators (H.P. and N.L.). Infections that could not be verified by the primary reviewer, in addition to a 10% random sample, were reviewed and confirmed independently by a panel of 5 infectious disease physicians (C.E., M.S., C.V., A.H.V., and J.T.M.). OHSU’s secure REDCap version 12.3.1 software was used to store all data collected through medical record review prior to data analysis.

### Patient comorbidities and potential risk factors

We collected patient demographic, comorbidity, and medical history data. We identified comorbidities including cardiovascular, chronic kidney, liver disease, and history of malignancy based on *International Classification of Disease, Tenth Revision* (ICD-10) coding associated with the hospital encounter and confirmed by chart review. We defined obesity as a body mass index >30 kg/m^2^. We performed manual chart review to collect the following: presence and type of malignancy, transfer from an outside facility, use and class of immunosuppressive agents prior to hospitalization and therapeutic use of remdesivir and corticosteroids for COVID-19 through the hospital encounter.

### Patient outcomes

Patient outcomes measured include all-cause mortality during the index hospitalization, total and intensive care unit (ICU) length of stay, and antibiotics that were ordered on the first 24 hours of hospitalization.

### Statistical Analysis

We calculated the cumulative incidence of secondary infections as the total frequency of proven or possible secondary pneumonia or bloodstream infection among all persons at risk. We described patient demographics, comorbidities, and other risk factors using summary statistical measures, and we have reported these measures stratified by secondary infection status. All covariates were considered for inclusion in a multivariable logistic regression model to identify possible risk factors of secondary infection (proven or possible). A stepwise model-building approach with a *P* < .05 entry and stay criteria was used to identify the final parsimonious multivariable logistic regression model; odds ratios (OR) and 95% confidence intervals (CIs) are reported. We calculated summary measures for all patient outcomes and stratified by secondary infection status. Because we hypothesized that missing data or misclassification may have been greater among patients transferred from outside facilities, we also performed a sensitivity analysis excluding transfer patients. All analyses were performed using SAS version 9.4 software (SAS Institute, Cary, NC).

## Results

**Fig. 1.**
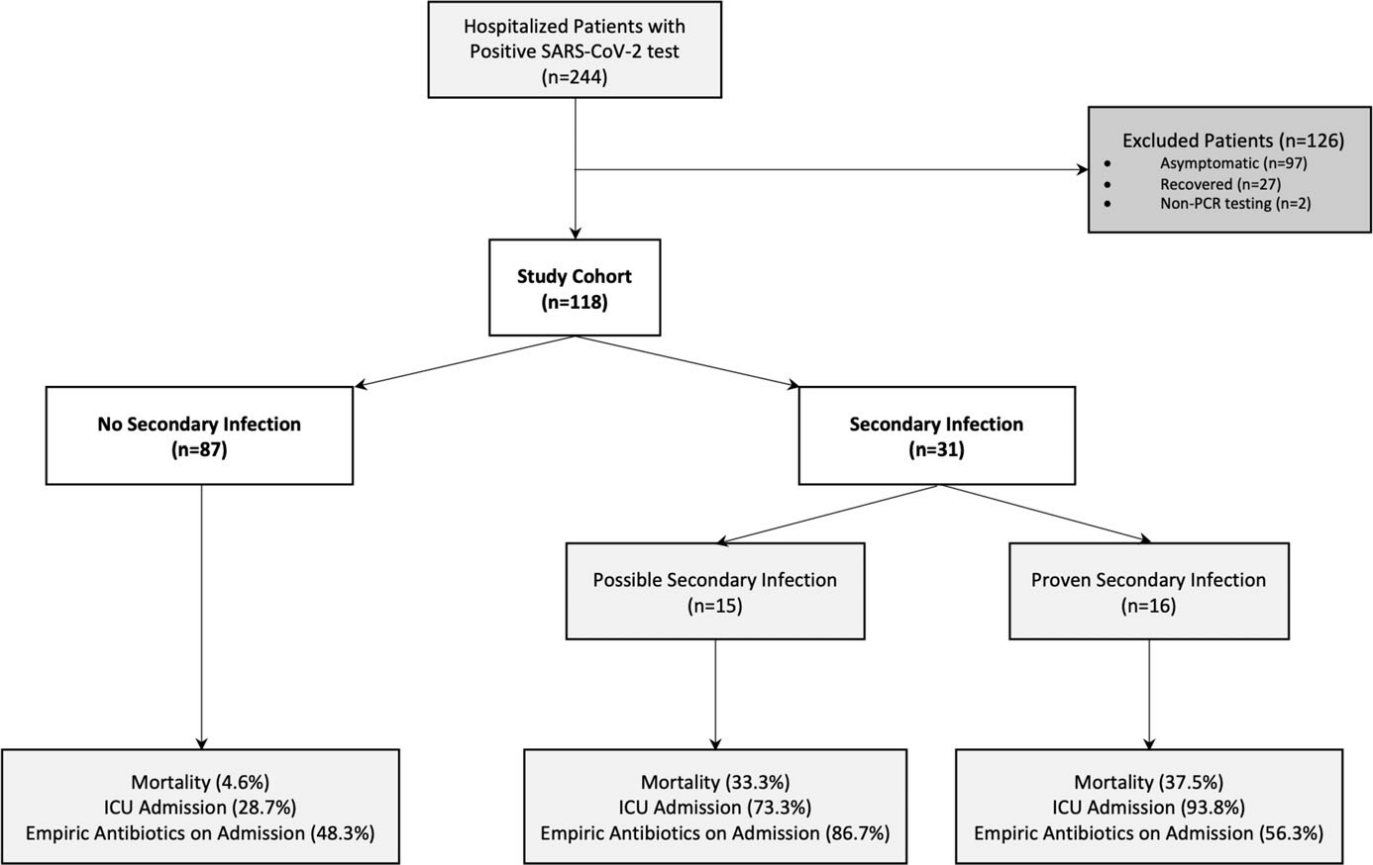
Study cohort identification and outcome frequencies. Proven secondary infections meet clinical, radiologic, and microbiologic criteria for infection. Possible secondary infection met only clinical and radiologic criteria. Note. ICU, intensive care unit.

We screened 244 encounters for patients with a positive SARS-CoV-2 PCR test for possible study inclusion (Fig. 1); 126 patient encounters were excluded, primarily due to asymptomatic COVID-19 and recovered cases (97 and 27 encounters, respectively). In total, 118 patients met inclusion criteria and were included in the study cohort. In total, 31 patients (26.3%) were identified with possible or proven secondary infection during their hospital stay. Among the 31 patients that developed secondary infections, 15 were classified as possible secondary infection and 16 were proven bacterial infections. No proven fungal infections were identified.

We observed a high prevalence of hypertension, hyperlipidemia, obesity, and diabetes among all groups (Table 1). A higher proportion of patient encounters among those with secondary infections had a history of immunosuppressant drug use prior to admission (9 of 31, 29.3%) compared to those whothat did not develop secondary infections (11 of 87, 12.6%). Among those with secondary infections on immunosuppressive agents, one-third were receiving anti-rejection medications. We observed a higher proportion of corticosteroid use for treatment of COVID-19 among those who developed a secondary infection (28 of 31, 90.3%) compared to those with no secondary infection identified (65 of 87, 74.7%). One case included prednisone used as a treatment for COVID-19 infection, whereas the remainder utilized dexamethasone in the treatment course. Those who developed a possible or proven secondary infection were also more likely to have been transferred from an outside facility (12 of 31, 38.7%) compared to those with no secondary infections (10 of 87, 11.5%). Only 1 patient in our study had received a single dose of COVID-19 vaccination, and no patients completed their primary series.

**Table 1.** Baseline Characteristics of Hospitalized Patients with COVID-19

Characteristics	Proven Secondary Infection (n = 16), No. (%)	Possible Secondary Infection (n = 15), No. (%)	No Secondary Infection (n = 87), No. (%)
Age, median y (IQR)	59.5 (53–67)	51 (44–69)	57 (42–69)
Sex, male	15 (93.8)	7 (46.7)	48 (55.2)
Nonwhite race	3 (18.9)	2 (13.3)	25 (28.7)
Transfer from outside	8 (50.0)	4 (27.7)	10 (11.5)
Hypertension	15 (93.8)	7 (46.7)	62 (71.3)
Hyperlipidemia	10 (62.5)	7 (46.7)	41 (47.1)
Coronary artery disease	7 (43.8)	5 (33.3)	24 (27.6)
Heart failure	6 (37.5)	1 (6.7)	23 (26.4)
Chronic kidney disease/ESRD	8 (50.0)	2 (13.3)	31 (35.6)
COPD	2 (12.5)	2 (13.3)	13 (14.9)
Smoking history	2 (12.5)	1 (6.7)	14 (16.1)
Alcohol use disorder	2 (12.5)	0	8 (9.2)
Cirrhosis	1 (6.3)	1 (6.7)	9 (10.3)
Obesity (BMI>30 kg/m^2^)	10/16 (62.5)	12/15 (80.0)	53/86 (61.6)
Diabetes	8 (50.0)	6 (40.0)	40 (46.0)
HIV	0	0	4 (4.6)
**Malignancy** ^**a**^			
Solid organ	0	2 (13.3)	3 (3.5)
Hematological	0	0	2 (2.3)
Immunosuppressant drugs^b^	5 (31.3)	4 (26.7)	11 (12.6)
Blood or sputum culture obtained	15 (93.8)	1 (6.7)	2 (2.3)
Vaccination initiated	1^c^		
**Treatments**			
Remdesivir	11 (68.8)	9 (60.0)	55 (63.2)
Corticosteroids^d^	15 (93.8)	13 (86.7)	65 (74.7)

Note. BMI, body mass index; IQR, interquartile range; ESRD, end-stage renal disease; COPD, chronic obstructive pulmonary disease; HIV, human immunodeficiency virus.

a Malignancy defined as active within the year prior to the index admission.

b Exposure >2 weeks of >20 mg prednisone, immunomodulatory agents: monoclonal antibodies such as tnf-α inhibitors, il6, rituximab, antirejection agents: cyclophosphamide, cyclosporin, tacrolimus. Chemotherapy use <6 months prior.

c Completed first dose of mRNA vaccination at the time of admission.

d Includes prednisone and dexamethasone.

Among the 31 encounters in which patients developed secondary infections, 15 were classified as possible secondary infection and 16 were proven infections (Table 2). The median time to onset of secondary infection was 5.5 days (IQR, 0.5–9.0) from admission for persons with proven secondary infections and 0 days (IQR, 0–0) for persons with possible infections. The majority met criteria for sepsis at the time of onset for both possible and proven infections at 12 (80.0%) of 15 and 14 (87.5%) of 16, respectively. There were overall more diffuse interstitial or ground glass patterns reported on imaging compared to focal consolidations with similar frequency between the 2 groups. However, the 2 groups diverged in oxygen delivery methods: patients with proven infections more frequently received mechanical ventilation or extracorporeal membrane oxygenation (ECMO) at the time of secondary infection onset. Patients without secondary infection had a low frequency of ECMO (1 of 87, 1.1%), mechanical ventilation (6.9% [6/87]), and BiPAP/high-flow nasal canula (10 of 87, 11.5%). Furthermore, possible infections were more frequently community onset (12 of 15, 80.0%), whereas most proven secondary infections were hospital acquired (12 of 16, 87.5%).

**Table 2.** Patient Characteristics at Onset of Secondary Bacterial Infections

Characteristic	Possible Secondary Infection (n = 15), No. (%)	Proven Secondary Infection (n = 16), No. (%)
Sepsis	12 (80.0)	14 (87.5)
**Oxygen delivery method**		
Nasal canula	2 (13.3)	0
Oxygen mask	3 (20.0)	2 (12.5)
Noninvasive ventilation (BiPAP/high-flow nasal canula)	6 (40.0)	1 (6.3)
Mechanical ventilation	3 (20.0)	8 (50.0)
ECMO	1 (6.7)	5 (31.3)
**Radiographic findings**		
Focal consolidation	5 (33.3)	4 (25.0)
Diffuse/ground glass opacity	10 (66.7)	10 (62.5)
Multifocal/nodular opacity	0	1 (6.3)
Cavitation	0	0
Pneumothorax	0	1 (6.3)
Transfer from outside facility	4 (26.7)	8 (50.0)
Community acquired	12 (80.0)	2 (12.5)
Hospital acquired	3 (20.0)	14 (87.5)
Days from admission to secondary infection onset, median (IQR)	0 (0–0)	5.5 (0.5–9.0)

Note. BiPAP, bilevel positive airway pressure; ECMO, extracorporeal membrane oxygenation; IQR, interquartile range.

Most secondary infections were bacterial pneumonias (13 of 16, 81.3%) diagnosed based on sputum samples, with 11 specimens obtained from endotracheal fluid aspiration and 1 from bronchoalveolar lavage (BAL) (Table 3). One infection was diagnosed by *Streptococcus pneumoniae* urine antigen detection. Also 5 patients had positive blood cultures. Among these 5, 1 patient had a primary bacteremia and 1 patient developed a central-line–associated bacteremia, both with coagulase-negative *Staphylococcus* spp. Furthermore, 3 cases of secondary bacteremia were identified: 1 case from a gastrointestinal source and 2 cases from secondary to bacterial pneumonia.

**Table 3.** Microbiologic Results Among 16 Cases With Confirmed Secondary Bacterial Infection

Microbiology Culture Characteristic	No. (%)
**Sputum culture**	12 (75.0)
Expectorated	0
Endotracheal aspirate	11 (91.7)
Bronchoalveolar lavage	1 (8.3)
**Blood Culture**	5 (31.3)
Primary	1 (20.0)
Central-line associated	1 (20.0)
Secondary	3 (60.0)
Bacterial pneumonia	2 (66.7)
Gastrointestinal	1 (33.3)
Urine antigen (*Streptococcus pneumoniae*)	1 (6.3)
Polymicrobial	1 (6.3)
Monomicrobial	15 (93.8)
**Bacterial species**	
Gram positive	10 (62.5)
*Staphylococcus aureus*	6 (60.0)
Methicillin susceptible	4 (66.7)
Methicillin resistant	2 (33.3)
Coagulase negative *staphylococcus* spp	2 (20.0)
*Streptococcus pneumoniae*	2 (20.0)
Gram negative	6 (37.5)
*Pseudomonas aeruginosa*	1 (16.7)
*Enterobacter cloacae*	1 (16.7)
*Escherichia coli*	2 (33.3)
*Klebsiella* spp	2 (33.3)
Anaerobe	1 (6.3)

The most common organism was *Staphylococcus aureus* isolated from 6 patients, 2 of which were methicillin resistant (Table 3). Other gram-positive organisms included coagulase-negative *Staphylococcus* spp as well as *Streptococcus pneumoniae*. Gram-negative organisms were isolated from 6 patients: 5 cultures were Enterobacterales group and 1 was *Pseudomonas aeruginosa*. One patient developed a polymicrobial infection.

Adverse patient outcomes (mortality, length of stay, and ICU) were higher among patients with secondary infection compared to those without (Table 4). Mortality was higher among patients with secondary infection at 35.5% (11 of 31) compared to 4.6% (4 of 87) among those with no secondary infection. A high proportion of empiric antibiotics were initiated on the day of admission at a rate of 71% for those with secondary infections and at a rate of 48.3% for those without secondary infections. Among those with secondary infections, most of those with possible secondary infections received ceftriaxone and azithromycin (both 66.7%), compared to those with proven secondary infection receiving cefepime with similar distribution of other antibiotic classes (Appendix Fig. 1 online). In our sensitivity analysis excluding patients transferred from an outside facility, a similar trend emerged regarding higher mortality and both ICU and total length of stay among those with secondary infections compared to those without (Appendix Table 1 online). Most groups with and without secondary infections still received empiric antibiotics on admission.

**Table 4.** Outcomes Among Patients With and Without Proven or Possible Secondary Infection

Outcome	No secondary Infection (n = 87)	Secondary Infection (n = 31)
In-hospital mortality, no. (%)	4 (4.6)	11 (35.5)
ICU admission, no. (%)	25 (28.7)	26 (83.9)
ICU length of stay, median d (IQR)	5 (2–11)	10.5 (8–19)
Total length of stay, median d (IQR)	9 (6–15)	19 (10–24)
Empiric antibiotics initiated <24 h of admission	42 (48.3)	22 (71.0)

Note. ICU, intensive care unit; IQR, interquartile range.

Our multivariable logistic regression model identified transfer from an outside hospital, use of immunosuppressant agents, and treatment with corticosteroids as significant independent risk factors for secondary infection (Table 5).

**Table 5.** Multivariable Logistic Regression Analysis for Factors Associated with Secondary Bacterial Infection (Possible or Proven)

Factor	Adjusted OR	95% CI
Transfer patient	5.34	1.89–15.03
Immunosuppressant Drugs	4.53	1.45–14.22
Dexamethasone	4.21	1.01–17.57

Note. OR, odds ratio; CI, confidence interval.

## Discussion

In this study, we observed an overall incidence of proven or possible secondary bacterial infections at 26.3% identified at various stages of hospitalization for COVID-19. Proven (ie, culture-confirmed) secondary infections were often hospital acquired (87.5%), with an incidence of 13.6%. In comparison, possible secondary infections were mostly community acquired (80%), with a similar incidence of 12.7%. Similar to other reports, culture-confirmed secondary bacterial infections with community onset were quite rare (2 of 118, 1.7%), and the majority of such infections were identified at later stages of hospitalization.^1–6,14^ We observed that clinically significant respiratory cultures occurred in patients with severe COVID-19 who were on mechanical ventilation; all of the specimens from proven secondary bacterial pneumonias were obtained from endotracheal aspirate or BAL. Such findings suggest that cases with severe COVID-19 are more susceptible to developing secondary bacterial pneumonia.

Severely ill COVID-19 patients are subjected to a high degree of intervention, placing them at risk for hospital-acquired bacteremia. Similar to other reports, bacteremia represented an overall minority of secondary bacterial infections among hospitalized patients (5 of 118, 4.3%) but results in significant morbidity and mortality.^15^ The higher mortality in patients with secondary infections (11 of 31, 35.5%) compared to those with no identified secondary infection (4 of 87, 4.6%) in this cohort was consistent with numerous prior studies.^1,4,6,7,9,14,16^

Similar to other studies, we observed a high proportion of individuals that received empiric antibiotics on admission.^1–5,8^ At the onset of the of the COVID-19 pandemic, providers’ experiences with influenza, coupled with a lack of available COVID-19 therapeutics, informed the decision to initiate antibiotics in patients with severe or worsening clinical status. Further studies are needed to evaluate antibiotic appropriateness to best inform antimicrobial stewardship and patient-level outcomes associated with antibiotic overuse.

Concordant with studies by Russell et al^14^ and Petty et al,^16^ *Staphylococcus aureus* was the most frequent organism isolated in our study (6 of 16, 37.5%). This finding contrasts with other studies that identified a predominance of *Acinetobacter baumanii* as well as *Pseudomonas aeruginosa* as the causal organism.^6,7,17^ These differences in the frequency of *S. aureus* can be due to environmental variability; such organisms are collectively accountable for hospital-associated infections with high antibiotic resistance and pathogenicity. Furthermore, significantly more multiclass-resistant and extended-spectrum–resistant *Pseudomonas* and Enterobacterales species have been isolated from clinical specimens during the COVID-19 pandemic compared to prepandemic periods.^18^

Our investigation identified several patients earlier in the hospital course who met criteria for possible secondary infection and received empiric antibiotics. In contrast to the cohort with proven infection, they had less need for mechanical ventilation, and presumably, limited access for reliable sputum sampling. In the setting of limited personal protective equipment and early questions regarding transmission, aerosol-generating procedures may have been avoided earlier during the pandemic, precluding sputum sampling. Because microbiologic data are scant in this setting, this is a challenging area for management because individuals requiring admission with COVID-19 resemble patients with bacterial pneumonia. Other studies have investigated various inflammatory markers: C-reactive protein, ferritin, and procalcitonin to help distinguish COVID-19 and community-acquired bacterial pneumonia.^19,20^ However, such control groups were compared to community-acquired bacterial pneumonia without COVID-19. Our institution does not have procalcitonin available; therefore, we were not able to evaluate its impact in our study. Future studies will need additional validation of such non–culture-based diagnostic strategies to help determine the presence or absence of secondary bacterial pneumonia, especially in early stages of COVID-19.

We identified that corticosteroid treatment for COVID-19 was independently associated with an increased likelihood of secondary bacterial infections, which is consistent with prior reports.^16,17^ Baseline use of immunosuppressive medications have also been reported with an increased likelihood of secondary infection.^21^ Finally, patients transferred from an outside facility were likely due to more severe COVID-19 because our institution serves as a referral center for the state of Oregon.

The standardized, multifaceted case definition for identifying secondary infections in COVID-19, which included clinical and radiologic criteria, serves as a major strength in this investigation. However, the study had several limitations. Data were retrospectively obtained and based on clinical practice, and cultures were only obtained if there was concern for a secondary bacterial infection. This can result in detection bias, resulting in underestimation of secondary infections among those without a high suspicion for secondary infection by the primary team during hospital admission. Patients transferred from outside facilities may be more likely to have missing data, which can introduce misclassification bias. We explored this possibility in our sensitivity analysis, in which transfer patients were excluded, yet similar results were observed suggesting minimal misclassification bias due to transfer patients. Steroid use for COVID-19 was seen throughout our cohort according to hospital treatment guidelines. Increased length of ICU stay and steroid use was seen with increased severity of illness, which was also associated with infection. This finding is not a surprising because patients with more complicated hospital courses and invasive procedures are at higher risk for hospital-acquired infections. Finally, the dynamic nature of the pandemic, which includes novel variants with varying pathogenicity and disease severity, may influence the incidence of secondary bacterial infections. Furthermore, the evolving landscape of COVID-19 therapeutics and vaccines attenuated disease severity for many patients and may also influence secondary infection risk.^22^ However, this must be balanced against the shifting immunity by ongoing vaccination efforts that serve as protective factors for severe disease. Our cohort largely represented a nonimmunized cohort prior to widespread availability of vaccines (Table 1). Ongoing research is needed to ensure the validity of our findings over the course of the pandemic.

In conclusion, culture-proven secondary infections in COVID-19 cases occurred among severe cases needing mechanical ventilation and were generally hospital acquired. Such cases manifested predominantly as secondary bacterial pneumonia with higher mortality compared to those with no identified secondary infections. A higher odds of immunosuppressant use at baseline and corticosteroids used for treatment of COVID-19 were observed among those who developed secondary infections.

## Supplementary material

For supplementary material accompanying this paper visit https://doi.org/10.1017/ice.2023.27. click here to view supplementary material
