# Supplementary material for: Incidence and risk factors for clinically confirmed secondary bacterial infections in patients hospitalized for coronavirus disease 2019 (COVID-19)
Source: Infect Control Hosp Epidemiol. 2023 May 15;44(10):1650–6. doi: 10.1017/ice.2023.27 (PMC10587378; doi:10.1017/ice.2023.27)
Supplement: Supplementary file 1 [file S0899823X23000272sup001.docx]

Appendix

Appendix Figure 1. Empiric Antibiotic within 24 Hours of Hospital Admission

Abbreviations: Azithromycin (AZI), Ceftriaxone (CTX), Piperacillin-Tazobactam (PIP), Cefepime (CEF), Vancomycin (VAN)

Appendix Table 1. Subgroup Analysis for Outcomes Excluding Transfer Patients

|  | No secondary infection  (N= 77) | Secondary infection  (N=19) |
| --- | --- | --- |
| In hospital mortality, n (%) | 3 (3.9%) | 8 (42.1%) |
| ICU admission, n (%) | 18 (23.4%) | 14 (73.7%) |
| ICU length of stay, median days (IQR) | 3.5 (2-5) | 10.5 (5-18) |
| Total length of stay, median days (IQR) | 7.5 (5-13) | 19 (8-22) |
| Empiric antibiotics received, n (%) | 48 (62.3%) | 15 (79%) |
